# BRAF: A Two-Faced Janus

**DOI:** 10.3390/cells9122549

**Published:** 2020-11-27

**Authors:** Pasquale Pisapia, Francesco Pepe, Antonino Iaccarino, Roberta Sgariglia, Mariantonia Nacchio, Gianluca Russo, Gianluca Gragnano, Umberto Malapelle, Giancarlo Troncone

**Affiliations:** Department of Public Health, University of Naples Federico II, 80131 Naples, Italy; pasquale.pisapia@unina.it (P.P.); francesco.pepe4@unina.it (F.P.); antiaccc@hotmail.com (A.I.); roberta.sgariglia@unina.it (R.S.); mariantonia.nacchio@unina.it (M.N.); gianlucar93@libero.it (G.R.); gianluca.gragnano@unina.it (G.G.); umberto.malapelle@unina.it (U.M.)

**Keywords:** predictive molecular pathology, prognostic biomarker, BRAF, molecular oncology

## Abstract

Gain-of-function of V-Raf Murine Sarcoma Viral Oncogene Homolog B (*BRAF*) is one of the most frequent oncogenic mutations in numerous cancers, including thyroid papillary carcinoma, melanoma, colon, and lung carcinomas, and to a lesser extent, ovarian and glioblastoma multiforme. This mutation aberrantly activates the mitogen-activated protein (MAP) kinase extracellular signal-regulated kinase (MEK)/extracellular signal-regulated kinase (ERK) signaling pathway, thereby eliciting metastatic processes. The relevance of *BRAF* mutations stems from its prognostic value and, equally important, from its relevant therapeutic utility as an actionable target for personalized treatment. Here, we discuss the double facets of *BRAF*. In particular, we argue the need to implement diagnostic molecular algorithms that are able to detect this biomarker in order to streamline and refine diagnostic and therapeutic decisions.

## 1. Introduction

The V-Raf Murine Sarcoma Viral Oncogene Homolog B (*BRAF*) oncogene, localized on chromosome 7q34, encodes for a serine–threonine protein kinase belonging to the rapidly accelerated fibrosarcoma (Raf) protein family (which also includes ARAF and CRAF proteins) [1]. Initially identified as a viral oncogene, the *RAF* gene was first described in 1983 [2]. Physiologically, Raf proteins and MAP kinase kinase kinases (MAPKKKs) play a key role in the mitogen-activated protein (MAP) kinase cascade [3]. In particular, they act as effectors of Rat sarcoma (Ras) proteins and as direct activators of the MAP kinase extracellular signal-regulated kinase (MEK)/extracellular signal-regulated kinase (ERK) pathway [4,5,6,7,8,9,10]. This pathway is associated with several cell functions, such as cell growth and proliferation, differentiation, migration, senescence, and apoptosis [11,12,13,14,15,16]. Generally, Raf proteins feature three conserved regions (CR), each having its own distinctive tasks. The CR1 domain is characterized by two subdomains: a Ras-binding domain (RBD), crucial for the interface with Ras proteins, and a cysteine-rich domain (CRD), necessary for the interaction with Ras proteins and Raf kinase domain auto-inhibition [17]. The CR2 domain works as an inhibitor against Ras protein binding and Raf activation [18]. Finally, CR3 features kinase activity [19].

**Figure 1:**
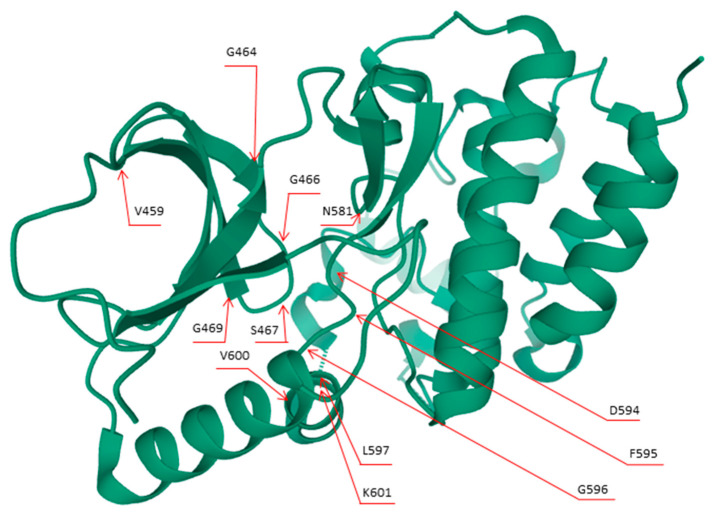
3D representation of the BRAF protein. The red arrows highlight the codons in which the main *BRAF* mutations arise. This figure was created using Mol* PDB ID Mol* and Research Collaboratory for Structural Bioinformatics (RCSB) Protein Data Bank (PDB) [32,33].

**Figure 2:**
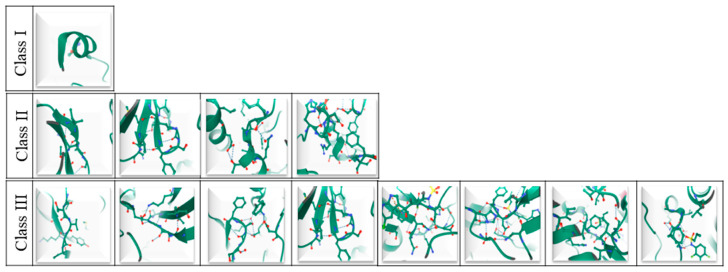
3D representation of BRAF mutations within the three-class system of classification. In particular from left to right: Class I p.V600; Class II p.G464, p. G469, p. L597, p.K601; Class III p.V459, p.G466, p.S467, p.G469, p.N581, p.D594, p.F595, p.G596. This figure was created by using Mol* PDB ID Mol* and Research Collaboratory for Structural Bioinformatics (RCSB) Protein Data Bank (PDB) [32,33].

The first evidence of an association between *BRAF* gene mutations and human cancers dates back to 2002 [20]. Generally, *BRAF* mutations are more frequently associated with human cancer than *ARAF* and *CRAF* alterations. As some studies suggest, this phenomenon may be due to the fact that whereas *BRAF* oncogenic activation is triggered by a substitution of a single amino acid, *ARAF* and *CRAF* oncogenic alterations require a double mutational event [21]. To date, *BRAF* mutations account for about 7% of all human solid tumors, with a high prevalence in papillary thyroid carcinomas (PTC), melanomas, colorectal cancers (CRC), and lung cancers [20,22,23,24,25,26,27]. The most common type of *BRAF* mutation is exon 15 p.V600E [20]. So far, about 200 *BRAF* mutant alleles have been described in human tumors [28]. Accordingly, *BRAF* mutations have been categorized into a three-class system according to their effect on the activity of BRAF protein (Table 1 and Figure 1 and Figure 2) [24,28,29,30,31].

Class I alterations are the most common ones. They include exon 15 p.V600 alterations. These mutations induce elevated levels of kinase activity, thereby promoting the activation of MEK/ERK pathways independently of Ras activation and protein dimerization [24,28,29,30,31]. Class II alterations are, instead, less common and involve several point mutations, in particular exon 11 p.G464E/V, exon 11 p. G469A/R/V, exon 15 p. L597Q/V, and exon 15 p.K601E/N/T, as well as gene fusions. Like class I, this class is Ras-independent. However, whereas Class I promotes only elevated levels of kinase activity, Class II triggers both intermediate and high kinase activity. Moreover, as opposed to Class I, Class II requires protein dimerization to activate the MEK/ERK pathway [24,28,29,30,31]. Finally, Class III alterations are associated with low or no kinase activity and require both Ras upstream activation and dimerization with CRAF to induce MER/ERK pathway activation; in addition, they commonly co-occur with upstream activating alterations such as Neuroblastoma RAS Viral Oncogene Homolog (*NRAS*) mutations, Neurofibromin 1 (*NF1*) loss, or receptor tyrosine kinase mutations [24,28,29,30,31].

Unlike Class II and Class III RAF-mutations, which may have less aggressive behavior, Class I mutations, namely, *BRAF* exon 15 p. V600, are associated with a higher degree of tumor aggressiveness and poor prognosis. Indeed, patients carrying this type of mutation have shorter disease-free survival (DFS) and bleaker overall survival (OS) rates than wild-type patients [34]. However, besides its prognostic role, accruing evidence has recently highlighted the role of *BRAF* exon p.V600 mutations, in particular *BRAF* exon 15 p. V600E, as predictive biomarkers of response to tyrosine kinase inhibitors (TKIs) [35,36,37].

In this review, we will examine the double facets of *BRAF* gene alterations in different tumor types to highlight the clinical relevance of this biomarker not only in improving the pathological assessment of human solid neoplasms but also in facilitating treatment decision-making and outcomes.

## 2. Prognostic Role of *BRAF* Mutations

### 2.1. Lung Cancer

The presence of *BRAF* mutations in non-small cell lung cancer (NSCLC) patients was first reported in 2011 [25]. Since then, subsequent studies have investigated the variable frequency of these alterations in NSCLC adenocarcinoma patients (from 1.5–3.5% to 7–8%) [31,38,39,40]. Regarding the subtypes of *BRAF* mutations, discordant results have been reported. For instance, whereas some authors highlight a higher prevalence of *BRAF* exon 15 p.V600E than *BRAF* non-p.V600E [25,41,42,43,44], others state opposite results [26,45,46]. Despite these contradictory findings, almost all NSCLC *BRAF*-mutated cases feature an adenocarcinoma morphology with a micropapillary growth pattern and strong expression of thyroid transcription factor 1 (TTF-1) [25]. Although *BRAF* mutations are strongly associated with glandular morphology, occasional reports have also described this alteration in small cell carcinoma and in different NSCLC subtypes, such as squamous cell carcinoma, large cell neuroendocrine carcinomas, and pulmonary sarcomatoid carcinomas [47,48,49,50]. Thus, it is conceivable that patients carrying *BRAF* mutations could be eligible to receive TKI treatments, even in the absence of an adenocarcinoma component.

From an epidemiological point of view, whereas some studies have indicated the higher frequency of *BRAF* mutations among females and current or former smokers [25,41], others have found no specific association between *BRAF* mutations and sex or tobacco smoking. Consequently, *BRAF* molecular testing by adopting next-generation sequencing (NGS) technologies in advanced-stage NSCLC patients is strongly recommended [51].

To date, the prognostic role of *BRAF* in NSCLC patients is still debated. Warth et al. showed a shorter disease-free survival (DFS) in *BRAF* exon 15 mutant patients than in wild-type patients [52]. Of note, they found no significant differences in the OS rates between the two groups [52]. A few years later, Zheng et al. reported contrasting results. Indeed, in their large-scale study aimed at identifying the prevalence of *BRAF* mutations among Chinese patients with lung adenocarcinoma, they found that the median relapse-free survival (RFS) of patients harboring either *BRAF* exon 11 or *BRAF* exon 15 was significantly longer than the RFS of NSCLC patients harboring other types of mutations, including Epidermal Growth Factor Receptor (*EGFR*), Kirsten Rat Sarcoma Viral Oncogene Homolog (*KRAS*), Anaplastic Lymphoma Kinase (*ALK*), Erb-B2 Receptor Tyrosine Kinase 2 (*ERBB2*), or wild-type (47.8 vs. 21.5 months) [53]. However, in a similar study, Paik et al. observed that the 2-year OS in patients harboring *BRAF* mutations was lower (57%) than that in patients harboring *EGFR* mutations (69%) and *ALK* rearrangements (91%). By contrast, it was higher in *KRAS*-mutated patients (40%) [43]. Similarly, Litvak et al. reported that the overall survival rate for patients with *BRAF* mutations was intermediate between those with *KRAS* (lower) and those with *EGFR* (higher) mutations [54].

On the other hand, several other studies have reported different OS rates. For instance, Kinno et al. described an insignificant difference between the OS rates of patients with *BRAF* exon 15 p.V600E and *BRAF* non-p.V600E mutations and wild-type patients [55]. Similarly, Villaruz et al. found no differences between the OS rates of *BRAF*-mutant patients and those harboring other genomic alterations [56]. Tissot et al., instead, highlighted a higher OS rate for *BRAF*-mutated NSCLC patients than for wild-type patients (22.1 vs. 14.5 months, respectively) [57]. Regarding the types of *BRAF* mutations influencing OS, the median OS was higher in *BRAF* exon 15 p.V600E mutants than in *BRAF* non-p.V600E mutants (25 vs. 13 months, respectively), even in stage IV patients (16 vs. 7 months, respectively) [57].

Clearly, the prognostic significance of *BRAF* mutations in lung cancer patients needs further elucidation. However, we are aware that planning prognostic perspective studies uninfluenced by BRAF-targeted therapies may be challenging, considering the burgeoning interest in *BRAF* as a predictive biomarker.

### 2.2. Melanoma

*BRAF* mutations occur in about 40–60% of melanomas [58]. A staggering 97% of these alterations are observed within exon 15 (codon 600) [59]. *BRAF* exon 15 is indeed the most common alteration, accounting for up to 90% of cases. It involves a T-to-A transversion in nucleotide 1799 (c.1799T>A), which, in turn, determines valine to glutamic acid substitution (p.V600E) [60]. Less frequently, a substitution from valine to lysine (p.V600K, 10–20%), arginine (p.V600R, 1%), methionine (p.V600M, 0.3%), or aspartic acid (p.V600D, 0.1%) may be observed within codon 600 [60]. Epidemiologically, *BRAF*-mutated melanomas occur in younger patients and very frequently display a superficial diffusion or nodular morphology. As opposed to wild-type cases, they are located in anatomical regions without chronic sun damage [61] and are more prone to metastasize to the brain than wild-type melanomas [23,62].

The prognostic role of *BRAF* in melanoma progression is not as controversial as in lung cancer patients. Broadly speaking, *BRAF*-mutated melanomas are characterized by more aggressive clinical features than wild-type ones [63]. Indeed, whereas a few studies report no significant differences in the survival rates of *BRAF*-mutated and wild-type metastatic melanoma patients [64,65], most highlight its negative prognostic factors [66]. For instance, Long et al. noted that although the time between disease presentation and metastatic progression is basically the same between *BRAF*-mutant and wild-type patients, a poorer median survival characterizes *BRAF*-mutant cases compared to wild-type patients (5.7 vs. 8.5 months, respectively) [61]. Still, Ekedahl et al. reported that *BRAF*-driven cases had a much worse prognosis than stage IV wild-types or *NRAS*-mutant melanoma patients [67]. As for OS, Moreau et al. reported that *BRAF* exon 15 p.V600 mutations correlated with poorer OS than wild-type cases (1.4 and 2.8 years, respectively) [68]. Likewise, Picard et al. and Barbour et al. demonstrated a negative prognostic role for *BRAF* mutations in stage III melanoma patients [69,70]. More recently, in a large systematic review and meta-analysis, Ny et al. confirmed the negative prognostic role of *BRAF* mutations in melanoma patients [71]. Moreover, *BRAF* seems to play a relevant prognostic role not only in the late stages but also in the earlier stages of the disease. For example, Cheng et al. found an association between *BRAF* mutations and poor prognosis in stage I and stage II melanoma patients [72]. Similarly, Nagore et al. reported poor prognosis in *BRAF*-driven localized melanomas [73].

### 2.3. Thyroid Cancer

The occurrence of *BRAF* mutation in thyroid cancer is even more complex to analyze. A large body of evidence indicates that the frequency of *BRAF* mutations (almost exclusively occurring as exon 15 p.V600E; from 18% to 87%) varies among thyroid cancers [74,75]. As a general rule, they occur more frequently in sporadic PTCs, in particular tall cell variant and aggressive microcarcinomas of adult patients [76,77], followed by PTC-derived anaplastic thyroid carcinomas (ATCs) and poorly differentiated carcinomas [74,78,79,80]. On the other hand, they are very rare or totally absent in other thyroid lesions, including medullary carcinomas, follicular carcinomas, and benign neoplasms [74,78,79,80]. In addition, our research group has demonstrated that *BRAF* exon 15 p.V600E point mutations play a diagnostic role in refining the risk of malignancy and treatment options in patients with cytological “indeterminate” nodules [81,82].

From a prognostic perspective, *BRAF*-mutated thyroid carcinomas feature more aggressive behavior and poorer outcomes than wild-types [77,83,84,85]. In addition, *BRAF* mutations have occasionally been associated with a higher risk of recurrence and persistence [83,86]. Consistently, Nikiforova et al. highlighted a significant association of *BRAF* mutations with extrathyroidal invasion and advanced stages at the time of diagnosis compared with wild-type cases [78]. Likewise, other clinical studies have reported a tight correlation between *BRAF* mutations and extrathyroidal diffusion [87,88,89]. In accordance with these findings, Elisei et al.’s 15-year follow-up study demonstrated the poorer prognostic outcome of *BRAF* exon 15 p.V600E-mutated patients compared to wild-types [77]. A few years later, Xing et al. carried out a retrospective multicenter study at 16 centers located in eight countries to investigate the role of the *BRAF* exon 15 p.V600E variant in PTC recurrence. Interestingly, their findings indicate that *BRAF* exon 15 p.V600E point mutation is a prognostic factor independent of the other conventional clinicopathological risk factors generally associated with PTC [90]. In addition, the authors emphasized that even in the early stages (stage I or II) of the disease and in micro-PTC, detection of *BRAF* exon 15 p.V600E is strongly associated with disease recurrence [90]. Similar results were also substantiated in another study by Elisei et al. [91]. Likewise, a significant negative correlation between *BRAF* exon 15 p.V600E point mutations and shorter OS rates was also emphasized in Liu et al.’s study [92].

Different lines of research have also investigated the epigenetic mechanisms whereby *BRAF* mutation impairs iodine metabolism. Indeed, *BRAF* exon 15 p.V600E point mutation promotes the downregulation and silencing of sodium-iodide symporter (*NIS*), a gene involved in iodine metabolism, by inducing histone deacetylation [93,94]. This phenomenon explains why *BRAF* exon 15 p.V600E thyroid carcinomas are resistant to radioiodine treatment [83]. Building on this evidence, Oler et al. demonstrated that the downregulation and silencing of thyroid iodide-metabolizing genes may also be associated with poorly differentiated tumors featuring more aggressive behavior [95]. They also indicated that the expression of *NIS* and Thyroid Stimulating Hormone Receptor (*TSHR*) is more significantly reduced in *BRAF*-mutated patients than in *BRAF* wild-types [95]. Finally, the authors advanced the hypothesis that this phenomenon might be responsible for additional genetic alterations, eventually leading to tumor aggressiveness and dedifferentiation [95].

### 2.4. Colorectal Cancer

*BRAF* mutations are much less frequent in CRC, accounting for only 10% of reported cases [96,97]. As for the other cancer types, the vast majority of *BRAF* mutations occur in codon 600 (exon 15 p.V600E) [98]. Epidemiologically, *BRAF*-mutated CRCs are detected more frequently in women and older patients (>70 years) [99]. Morphologically, *BRAF*-mutated CRCs are primarily located in the proximal colon (right side) and show mucinous, serrated, and poorly differentiated histology [99,100]. Concerning the subtypes of *BRAF* mutations, non-p.V600E mutations seem to occur more frequently in men and younger patients, have low-grade histology, and are localized on the distal colon site, in contrast to *BRAF* exon 15 p.V600E mutations [101]. In addition, patients harboring *BRAF* non-p.V600E show a longer median OS than *BRAF* exon 15 p.V600E-mutated patients [101]. Epigenetically, *BRAF*-mutated CRCs feature a high CpG island methylator phenotype (CIMP-H) and high microsatellite instability (MSI-H) [102,103].

Regarding its impact on prognosis, CRC patients with BRAF exon 15 p.V600E mutations show lower OS, DFS, and cancer-specific survival (CSS) rates than wild-type patients, despite the stage of the disease (II or III) or the adoption of chemotherapeutic regimens after surgery [104]. Consistently, Roth et al. reported lower OS rates for *BRAF*-mutated patients than for wild-types [105]. Samowitz et al. highlighted that *BRAF* exon 15 p.V600E mutant CRC patients have a worse prognosis in terms of OS regardless of age, stage (II to IV), and tumor site compared to *BRAF* wild-type patients [98]. Notably, the authors also observed that *BRAF* exon 15 p.V600E mutant CRC patients featuring microsatellite-stable (MSS) tumor patterns had significantly lower OS rates than MSI tumors, regardless of the stage of the disease (II to IV) [98]. Conversely, *BRAF* mutations did not influence the better prognosis of MSI CRCs [98]. Similar results have been reported by Ogino et al. [106]. In their experience, the authors observed that *BRAF*-mutated CRC patients were characterized by worse outcomes than *BRAF* wild-type cases [106]. When considering both *BRAF* and MSI status, they observed the worst OS rate in *BRAF*-mutated and MSS CRC patients [106]. Interestingly, *BRAF* mutations only slightly affected the outcome of MSI-H tumors [106].

The negative prognostic role of *BRAF* mutations has long been established in the metastatic setting. Back in 2009, findings from FOCUS, a large, multicenter clinical trial, indicated that although *BRAF* mutation did not significantly affect progression-free survival (PFS), it did have a worse impact on the OS of patients, compared with wild-types [107]. Conversely, in a pooled analysis of four clinical trials (FOCUS, CAIRO, CAIRO2, and COIN), both median PFS and median OS rates were lower in *BRAF*-mutant patients than in wild-types [108]. In addition, the authors further observed that proficient mismatch repair (pMMR) of CRC tumors harboring *BRAF* mutations had a lower median PFS and OS than *BRAF* wild-types [108]. Another broad pooled analysis, assessing the independent negative prognostic role of *BRAF* mutations in OS, revealed that *BRAF*-mutant patients had a worse prognosis even after metastasectomy [109]. In accordance with these studies, several others have confirmed worse outcomes in *BRAF*-mutated patients after lung or liver metastasis surgical resections than in wild-types [110,111,112].

### 2.5. Other Lesions

Besides the high incidence of *BRAF* mutants in the neoplasms mentioned above, other less common entities may also feature *BRAF* mutations. Among these are pediatric low-grade gliomas (PLGGs). These lesions are highly frequent among pediatric patients harboring *BRAF* p.V600E point mutations and have poorer outcomes than wild-type patients [113,114]. Conversely, glioblastoma patients harboring *BRAF* exon 15 p.V600E have the same prognosis as wild-type patients, despite the prevalence of epithelioid morphology without isocitrate dehydrogenase (*IDH*) alterations [115].

In breast cancer patients, the prognostic scenario is very similar, albeit the frequency of these aberrant mutations is low. Generally, poorer prognosis is observed in triple-negative breast cancers [115]. In addition, *BRAF* gene alterations may be a rare cause of anti-human epidermal growth factor receptor 2 (HER2) therapy resistance [116].

Finally, *BRAF* mutations have also been detected in ovarian cancer. Indeed, Grisham et al. rather recently observed that *BRAF* mutations had better prognostic outcomes in early-stage low-grade serous ovarian cancer. The authors hypothesized that the better OS rates in these patients were due to the possibility that *BRAF* mutations in patients with serous borderline disease prevent progression to more aggressive stages [117].

## 3. Predictive Role of *BRAF* Mutations

### 3.1. Lung Cancer

Besides having a prognostic role, *BRAF* mutations have also emerged as positive predictive markers for identifying NSCLC patients who might benefit from the administration of targeted therapy [118,119,120,121]. Considerable evidence has demonstrated that BRAF inhibitors, namely, vemurafenib and dabrafenib, effectively work against *BRAF* exon 15 p. V600-positive tumor cells [122].

In a large study in which vemurafenib was administered to pretreated nonmelanoma *BRAF* exon 15 p.V600E-mutated patients, an objective response was observed in 42% of NSCLC patients, with a median PFS of 7.3 months and a 12-month PFS rate of 23% [123]. Consistently, the results of a vemurafenib basket (VE-BASKET) trial, involving *n* = 62 NSCLC patients, recently confirmed not only the efficacy of this *BRAF* inhibitor but also its safety profile in advanced-stage NSCLC *BRAF* exon 15 p.V600-positive patients [124]. Of note, vemurafenib also shows promising results in brain metastases [120].

The other BRAF inhibitor, dabrafenib, appears to be just as promising as vemurafenib. In a phase I dose-escalation trial, dabrafenib showed safety and efficacy in different solid tumors harboring *BRAF* exon 15 p.V600 point mutations, including NSCLC patients [125]. Noteworthy, in one study, dabrafenib was successfully adopted when a *BRAF* exon 15 p.V600E-mutated lung adenocarcinoma patient became resistant to vemurafenib [121]. In accordance with these studies, Gautschi et al. demonstrated the efficacy of different targeted therapies (vemurafenib, dabrafenib, sorafenib) in advanced-stage NSCLC patients harboring *BRAF* mutations [126]. They observed that the OS of patients receiving targeted therapies was longer in patients harboring a *BRAF* exon 15 p.V600E mutation than in those harboring non-p.V600E mutations (25.3 vs. 11.8 months) [126].

The efficacy of dabrafenib as monotherapy, or in combination with other drugs, has also been investigated in a phase-II clinical trial involving 84 *BRAF* (exon 15 p.V600E)-positive NSCLC patients. Patients were divided into two cohorts: Cohort A received dabrafenib alone [127], whereas Cohorts B and C received dabrafenib in combination with the MEK inhibitor trametinib [128,129]. On the whole, the objective response and disease control rates observed in Cohort A were 33% and 58%, respectively. In addition, the median PFS and median duration of response (DOR) were 5.5 and 9.6 months, respectively [127]. In Cohorts B and C, dabrafenib, together with trametinib, was administered to previously treated and untreated advanced-stage NSCLC patients harboring the *BRAF* exon 15 p.V600E point mutation [128,129]. In both cohorts, the combination showed clinically relevant antitumor activity in terms of overall responses (63.2% and 64.0%, respectively), PFS (8.6 and 14.6 months, respectively), DOR (9.0 and 15.2 months, respectively), and a manageable safety profile [128,129]. In addition, although the data were limited, the combination of these two inhibitors was able to act on brain metastasis [127,128,129]. After these promising results, the combination of dabrafenib and trametinib was approved by the Food and Drug Administration (FDA) and the European Medical Agency (EMA) for advanced-stage NSCLC patients harboring a *BRAF* exon 15 p.V600E point mutation, irrespective of previous treatments [130,131].

Regarding other types of BRAF inhibitors, anecdotal experiences have reported the efficacy of the multitarget kinase inhibitor sorafenib. Indeed, this inhibitor has a broad spectrum efficacy by acting on several types of kinases in *BRAF* non-p.V600-mutated patients (exon 11 p.G469R and exon 11 p.G469V). Among these are BRAF, CRAF, KIT Proto-Oncogene, Receptor Tyrosine Kinase (c-KIT), Fms Related Receptor Tyrosine Kinase 3 (FLT-3), Rearranged During Transfection (RET), Vascular Endothelial Growth Factor Receptor 2 (VEGFR-2), VEGFR-3 and Platelet-Derived Growth Factor Receptor Alpha (PDGFRA) in *BRAF* non-p.V600-mutated patients (exon 11 p.G469R and exon 11 p.G469V) [132,133]. Finally, two phase-I clinical trials demonstrated the efficacy and safety of ERK inhibitors (e.g., ulixertinib, an ERK1/2 kinase inhibitor) and pan-RAF inhibitors (e.g., LY3009120) in *BRAF*-mutant patients [134,135].

### 3.2. Melanoma

Like in lung cancer, *BRAF* mutations, in particular, exon 15 p. V600 point mutations, feature a positive predictive role in melanoma patients for the administration of TKIs [136]. In the BRIM-3 randomized, phase III clinical trial, the effect of vemurafenib on PFS and OS rates was compared with standard chemotherapy treatment (dacarbazine) in advanced-stage previously untreated melanoma patients harboring a *BRAF* exon 15 p.V600E point mutation [137]. Overall, patients treated with vemurafenib experienced a significant improvement in PFS, treatment response, and OS rates compared with chemotherapy-treated patients, with a tolerable profile of toxicity [137]. Likewise, in an extended follow-up analysis on the overall population of the BRIM-3 clinical trial, the authors confirmed the efficacy of vemurafenib on OS rates in advanced-stage previously untreated melanoma patients harboring *BRAF* exon 15 p.V600E or other less common exon 15 p.V600 point mutations compared with dacarbazine [138]. In another phase III, randomized, controlled clinical trial, in which the efficacy of dacarbazine was compared with the BRAF inhibitor dabrafenib, similar results were obtained in terms of PFS and OS in advanced-stage previously untreated melanoma patients harboring *BRAF* exon 15 p.V600 point mutations (p.V600E and p.V600K) [139,140].

As for lung cancer, several combination therapies have been investigated. One approach entails the combination of the BRAF inhibitor dabrafenib with the MEK inhibitor trametinib. In advanced-stage previously untreated melanoma patients harboring *BRAF* exon 15 p.V600 point mutations (p.V600E and p.V600K), such a combination significantly improved PFS (11.0 versus 8.8 months, respectively), OS (25.1 versus 18.7 months, respectively), and overall response rates (69.0% versus 53.0%, respectively) compared with dabrafenib alone [141,142,143,144]. Similar results have been reported for the association of BRAF inhibitor dabrafenib with the MEK inhibitor trametinib with respect to the BRAF inhibitor vemurafenib alone in terms of PFS (11.4 versus 7.3 months, respectively), objective response rate (ORR; 64.0% versus 51.0%, respectively) and 12-month OS (72.0% versus 65.0%) [145]. Another combination approach, adopted in the coBRIM clinical trial, involved the administration of the BRAF inhibitor vemurafenib and the MEK inhibitor cobimetinib [146]. Even in this case, the combination of the two drugs significantly improved OS and PFS rates in previously untreated advanced-stage *BRAF* exon 15 p.V600-mutated melanoma patients treated with the combination therapy compared with vemurafenib plus placebo (control group) [146]. Remarkably, no significant high-grade (3 or higher) adverse events were reported in the combination group [146]. Another valid combination approach is represented by the association of BRAF inhibitor encorafenib and MEK inhibitor binimetinib. In the COLUMBUS phase III clinical trial, previously treated or untreated advanced-stage melanoma patients harboring *BRAF* exon 15 p.V600E or p.V600K point mutations were randomly assigned to receive the combination regimen or single BRAF inhibitor therapies (vemurafenib or encorafenib). Overall, the combination strategy demonstrated, with respect to encorafenib or vemurafenib administration alone, a higher PFS (14.8 versus 9.2 versus 7.3 months, respectively), overall response rate (63.0% versus 51.0% versus 40.0%, respectively) and DOR (18.6 versus 15.2 versus 12.3 months, respectively) [147]. Interestingly, the association of BRAF and MEK inhibitors demonstrated a higher PFS (8 versus 3.7 months, respectively) and OS (17.3 versus 7.3 months, respectively) with respect to BRAF inhibitors alone in patients harboring rare BRAF non-p.V600 point mutations [148].

Still, another approach in *BRAF*-mutated advanced-stage melanoma patients is a combination of TKIs and immunotherapies. In murine models, in fact, TKIs favor immunotherapy activity by increasing tumor-infiltrating lymphocytes [149]. Nevertheless, severe toxicities have been reported for the association of ipilimumab (an anti-Cytotoxic T-Lymphocyte Antigen 4 (CTLA-4) monoclonal antibody) and TKIs [150,151].

Promising results and manageable toxicities have been observed with concurrent administration of three agents: (1) durvalumab (anti-Programmed Death-Ligand 1 (PD-L1)) or pembrolizumab (anti-PD-1) plus dabrafenib plus trametinib [35]; (2) atezolizumab (anti-PD-L1) plus cobimetinib (MEK inhibitor) plus vemurafenib (BRAF inhibitor) [152]. Based on the results of the phase-III clinical trial IMspire 150, the FDA approved the use of the latter triple combination [153,154] for advanced-stage melanoma patients harboring *BRAF* exon 15 p.V600 point mutations. Conversely, the COMBI-I phase III clinical trial did not meet the primary end-point (PFS). In this study, the association of the anti-PD-1 spartalizumab plus dabrafenib plus trametinib did not significantly increase the PFS with respect to the association of dabrafenib plus trametinib [155].

### 3.3. Other Lesions

The efficacy of the combination strategy with debrafenib (BRAF inhibitor) and trametinib (MEK inhibitor) was strongly demonstrated in NCI-MATCH Trial Subprotocol H. Overall, in this study, which enrolled patients with different cancer types harboring *BRAF* exon 15 p.V600 mutations, an ORR of 38% was reached [156].

Regarding thyroid cancers, radioiodine treatment represents an adjuvant approach for early-stage patients undergoing thyroidectomy, and it is the most important therapeutic choice for advanced-stage patients [157]. However, as previously reported, *BRAF* exon 15 p.V600E-mutated thyroid carcinomas are resistant to the radioiodine treatment approach [83]. Nevertheless, these patients may benefit from targeted treatments. More recently, the efficacy of BRAF inhibitors vemurafenib for advanced-stage *BRAF* exon 15 p.V600E-mutated thyroid cancer patients refractory to radioiodine treatment [158] and dabrafenib for *BRAF* exon 15 p.V600E-mutated metastatic PTC patients has been demonstrated [159]. However, despite FDA approval, the European Medicines Agency (EMA) did not extend the use of vemurafenib and dabrafenib for the treatment of thyroid cancer patients due to the unfavorable toxicity profile [160]. Conversely, the combination of the BRAF inhibitor dabrafenib and the MEK inhibitor trametinib showed promising results and tolerability in *BRAF* exon 15 p.V600E-mutated unresectable or metastatic anaplastic thyroid carcinoma patients and obtained FDA approval [161]. In fact, Subbiah et al. reported, in these patients without any other available treatment choice, an overall response rate of 69% demonstrates a meaningful therapeutic advance for this previously untreatable disease [162].

The combination strategy approach seemed to be more effective in CRC patients harboring a *BRAF* exon 15 p.V600E point mutation with respect to single-agent regimens. In the BEACON CRC phase III clinical trial, metastatic CRC patients harboring a *BRAF* exon 15 p.V600E point mutation, with disease progression after one or two previous treatment regimens, were randomized to receive encorafenib (BRAF inhibitor), binimetinib (MEK inhibitor), and cetuximab (anti-EGFR) or encorafenib and cetuximab, or other treatment approaches, including cetuximab and irinotecan or cetuximab and FOLFIRI (folinic acid, fluorouracil, and irinotecan). Overall, the median OS was 9.0, 8.4, and 5.4 months, respectively. Noteworthy, median PFS was higher in the triple-combination group (4.3 months) and in the association of encorafenib and cetuximab group (4.2 months) with respect to the other group (1.5 months). Similar promising results were reported for ORR (26.0%, 20.0%, and 2.0%, respectively). These data demonstrated the clinical efficacy of the combination strategy (encorafenib, binimetinib, and cetuximab) in previously treated metastatic CRC patients harboring a *BRAF* exon 15 p.V600E point mutation [163].

The combination strategy, with the BRAF inhibitor dabrafenib plus MEK inhibitor trametinib, demonstrated promising efficacy in patients with recurrent or refractory *BRAF* exon 15 p.V600E-mutated high-grade and low-grade gliomas. Overall, ORRs of 27.0% and 56.0% were reached [164].

## 4. Conclusions

*BRAF* mutations are reported in about 7% of solid tumors, with a high prevalence in PTC, melanoma, colorectal cancer, and lung cancer [20,22,23,24,25,26,27]. Located at codon 600 of exon 15, most of these mutations result in an amino acid substitution of valine to glutamic acid (p.V600E) [20]. Overall, about 200 *BRAF* mutant variants have been described in human tumors [28]. Like the two-faced mythological deity Janus, *BRAF* mutations are double-faceted, having both a prognostic and a predictive role. In fact, although *BRAF* mutations are typically associated with a negative prognostic role especially in, lung cancer, melanoma, thyroid, and CRC, their positive predictive role has recently emerged in melanoma, lung cancer, thyroid carcinoma, CRC, and other cancers, such as glioma patients. For this reason, implementing *BRAF* mutational analysis, alongside other clinically relevant gene alterations, is pivotal in selecting patients for targeted treatments. Noticeably, our research team at the Predictive Molecular Laboratory (Department of Public Health, University of Naples Federico II, Naples, Italy) has designed, developed, and validated a narrow NGS custom panel, named SiRe^®^, that is able to cover 568 mutations in six genes of clinical interest involved in four solid tumors: NSCLCs, CRCs, melanomas, and gastrointestinal stromal tumors. The six genes of interest include *BRAF*, *EGFR*, *KRAS*, *NRAS*, *KIT*, and *PDGFRA*. Moreover, we subsequently made this analysis even more efficient by optimizing the workflow so as to enable sequencing in both tissue and liquid biopsy specimens [26,165,166,167,168,169,170,171]. Alternatively, researchers in our lab and in other groups have also proposed more rapid diagnosis by adopting automated real-time polymerase chain reaction (RT-PCR) technologies [172,173,174,175]. Taking a further step, we have recently demonstrated the possibility of genotyping *BRAF* mutation directly from thyroid fine-needle aspiration (FNA) rinses to enable interventional cytopathologists to perform rapid molecular on-site evaluation (ROME) [172].

In conclusion, this literature review has highlighted the Janus-faced nature of *BRAF* mutations. Indeed, besides its well-documented prognostic role, *BRAF* mutations are emerging as crucial predictive markers in personalized cancer therapy. The relevance of *BRAF* as a new predictive marker lies in the fact that cancer patients harboring this mutation show poor response rates to conventional therapies. Therefore, correct identification of *BRAF* mutations, together with other types of aberrant molecular markers, is key to selecting patients for target treatments. Notably, as opposed to standard chemotherapy, a non-negligible percentage of *BRAF*-mutated cancer patients are benefitting from BRAF and/or MEK inhibitors and/or immunotherapy (anti-CTLA-4, -PD-1, -PD-L1) in terms of OS and tolerable toxicity. However, the efficacy, as well as safety profiles, of these treatments may vary among the different subsets of mutant *BRAF* tumors. For this reason, we adamantly support the application of molecular predictive laboratory devices that are able to cover at least *BRAF* exon 15 p.V600 clinically relevant mutations.

## Figures and Tables

**Table 1:** *BRAF* mutations classification system.

Class I	Class II	Class III
p.V600D/E/K/M/R	p.G464E/V; p. G469A/R/V; p. L597Q/V; p.K601E/N/T; gene fusions	p.D287H; p.V459L; p.G466A/E/V; p.S467L; p.G469E; p.N581I/S; p.D594A/G/H/N; p.F595L; p.G596D/R

Abbreviation: *BRAF*, V-Raf Murine Sarcoma Viral Oncogene Homolog B.
